# Incorporating of an enhanced radiation protection device into the treatment of ST elevation myocardial infarctions: A single center retrospective analysis

**DOI:** 10.1016/j.ahjo.2026.100816

**Published:** 2026-06-19

**Authors:** Jennifer Hutchings, Eve Mai, Stephen P. Blay, Chelsea Ballinger, Robert F. Riley

**Affiliations:** a Overlake Medical Centers & Clinics, United States of America

**Keywords:** STEMI, Radiation, Door-to-balloon time, Orthopedic, Injury

## Abstract

**Study objective:**

To evaluate whether incorporation of the EggNest Complete enhanced radiation protection device (ERPD) affects door-to-balloon (D2B) time in treatment of patients presenting with ST-elevation myocardial infarction (STEMI).

**Design:**

Single-center retrospective observational study.

**Setting:**

Catheterization laboratories in a tertiary care hospital.

**Participants:**

133 consecutive patients presenting with STEMI (71 in the ERPD group, 62 in the standard shielding) group from July 1, 2024 until December 31, 2025.

**Interventions:**

Evaluation of D2B time components in rooms with ERPD vs standard shielding.

**Main outcome measure:**

D2B time (total time and time-based components).

**Results:**

There was no statistically significant difference in overall D2B times between groups (48.0 vs 52.0 min; *p* = 0.32) or it's individual components. Use of the EggNest Complete ERDP was not associated with D2B (β = 1.14, *p* = 0.88).

**Conclusion:**

Use of the EggNest Complete ERPD does not delay STEMI reperfusion. These findings support a paradigm shift from operator-dependent shielding to integrated, system-level radiation protection, demonstrating that improvements in occupational safety for cath lab workers can be achieved without tradeoffs in the timeliness of life-saving reperfusion therapy.

## 1 Introduction

Door-to-balloon (D2B) time remains a central quality metric in the management of patients with ST-elevation myocardial infarction (STEMI) undergoing primary percutaneous coronary intervention (PCI) [1]. Current guidelines recommend timely reperfusion with a target D2B time of ≤90 min, while European Society of Cardiology guidelines emphasize minimizing system delays, with targets approaching ≤60 min when feasible [2], [3]. Prior studies have consistently demonstrated that shorter D2B times are associated with improved clinical outcomes, including reductions in mortality, underscoring the importance of optimizing each component of the STEMI care pathway [4], [5], [6], [7]. As a result, ongoing efforts have focused on identifying workflow and system-level factors that may influence treatment delays and opportunities for quality improvement [8], [9], [10].

Radiation exposure remains a significant occupational hazard for operators and staff performing fluoroscopically guided cardiovascular procedures, including patients presenting with STEMI. Chronic exposure has been associated with increased risks of cataracts, malignancy, and other adverse health effects [11]. Although lead-based personal protective equipment is the current standard for radiation mitigation, its prolonged use is associated with substantial musculoskeletal strain. High rates of orthopedic injury among interventional cardiologists have been well documented, with many practitioners experiencing chronic pain, procedure-related limitations, or early retirement related to occupational injury. In contemporary surveys, a majority of operators report work-related musculoskeletal issues, including spine and joint pathology, highlighting the need for improved protective strategies within the catheterization laboratory [12], [13].

Enhanced radiation protection devices (ERPD) have emerged as a promising solution, offering significant reductions in operator radiation exposure while alleviating the physical burden associated with traditional lead shielding [14], [15], [16]. However, integration of these systems into established cardiac catheterization laboratory (CCL) workflows could introduce potential concerns regarding procedural setup, access, and efficiency—particularly in time-sensitive clinical scenarios such as primary PCI for STEMI.

Given the critical importance of minimizing delays in reperfusion during the treatment of STEMI, it is essential to determine whether implementation of ERPDs affect D2B times compared to traditional shielding methods. We performed a single-center retrospective analysis aimed at evaluating the impact of incorporating the EggNest Complete ERPD into the CCL on D2B times in consecutive patients presenting with STEMI compared to CCLs with traditional standard shielding.

## 2 Materials and methods

A retrospective analysis of the medical records at a single site was performed over an 18-month period from July 1, 2024 through December 31, 2025, after installation of an EggNest Complete ERPD in one of its CCLs. The other two CCLs at the institution utilized standard shielding instead of the EggNest Complete system. STEMIS are treated in all three rooms, with room choice typically being randomly distributed between the three rooms. All labs in this study used Philips Azurian c-arms; there were no significant differences between the rooms except for the presence or absence of an ERPD. Study groups were defined based on whether the STEMI case was performed in the CCL with the EggNest Complete ERPD versus in the other two CCLs with standard shielding. Baseline demographics, case details, and D2B times, including each component of the D2B times, were collected for each group. Of note, D2B time components have been routinely collected at the study site for over a decade as part of a continuous quality improvement program.

The EggNest Complete ERPD is a comprehensive passive radiation protection platform. It is comprised of a carbon-fiber base platform with integrated 0.5 mm non‑lead equivalent shielding that replaces the traditional table mattress. Hanging Flex Shields below the table and flip shields above the table passively protect staff and physicians in any location around the room. The flex shields are retractable, allowing for full C-arm rotations when needed. A ceiling- or boom-mounted clear acrylic shield (the Complete shield) with 1.0 mm lead equivalent shielding is placed over the patient, such that a cutout with a radiation shielding fringe is placed against the patient and extends across the arm. The right arm is held in a cradle with additional radiation shielding for radial cases.

The standard shielding group consists of rooms with two shields positioned between the patient and operating physician: a ceiling-mounted upper body lead shield with a patient contour cutout and a lower body lead shield attached to the side of the operating table extending from table to floor.

### 2.1 Statistical analysis

Continuous variables were summarized as medians with interquartile ranges (IQRs) given the non-normal distribution of time interval data. The door-to-balloon pathway was deconstructed into sequential time intervals, including door-to-electrocardiogram (EKG), EKG-to-activation (CCL staff page), patient readiness for the CCL in the emergency department (ED), transport from the ED to the catheterization laboratory, arrival in the CCL to procedural start, and procedural start to first device deployment. Each interval was derived from recorded time stamps as the difference between consecutive workflow steps. Total D2B time was defined as the interval from ED arrival to first device activation.

Comparisons between the EggNest Complete CCL and standard shielding CCL groups were performed using the Mann–Whitney *U* test for all continuous variables. To evaluate independent predictors of door-to-device (D2D) time, a multivariable linear regression model was constructed with D2B time (minutes) as the dependent variable. The primary exposure of interest was procedure room assignment, categorized as the EggNest Complete room versus all other (standard shielding) rooms. Additional covariates were selected a priori based on clinical relevance and included: vascular access site (femoral vs radial), presence of cardiogenic shock prior to procedural start, and requirement for mechanical ventilation (intubation) before or during the procedure.

Categorical variables were coded as binary indicators (e.g., femoral access = 1, radial access = 0; shock = yes/no; intubation = yes/no). Continuous variables were assessed for normality and retained in their original scale. Observations with missing data for variables included in the model were excluded using complete-case analysis.

Linear regression assumptions, including linearity, independence, and homoscedasticity of residuals, were assessed. Regression coefficients (β) with corresponding 95% confidence intervals (CIs) and *p*-values were reported. A two-sided p-value <0.05 was considered statistically significant. Statistical analyses were performed using Python (pandas, NumPy, and SciPy libraries).

This study was approved by the site's offsite IRB.

## 3 Results

A total of 133 continuous patients presented with ST-elevation myocardial infarction (STEMI) undergoing primary percutaneous coronary intervention during the study period and were included in the analysis, including 71 treated in the CCL with an EggNest Complete ERPD and 62 treated in CCLs using standard shielding.

Baseline demographic and clinical characteristics are summarized in Table 1. The median age was similar between groups, and there were no significant differences in sex distribution or cardiovascular risk factors. Overall, the two cohorts were well balanced with respect to baseline clinical characteristics.

**Table 1:** Study groups demographics.

Variable	EggNest Complete (*n* = 71)	Standard Shielding (*n* = 62)	p-value
Age	66.0 [55.8–74.0]	65.0 [55.0–76.0]	0.919
Male gender	50 (69.4%)	49 (80.3%)	0.74
Prior MI	10 (13.9%)	11 (18.0%)	0.679
Prior PCI	13 (18.1%)	11 (18.0%)	1.000
Prior CABG	4 (5.6%)	2 (3.3%)	0.833
History of hypertension	42 (58.3%)	36 (59.0%)	1.000
History of dyslipidemia	43 (59.7%)	36 (59.0%)	1.000
History of diabetes	14 (19.4%)	16 (26.2%)	0.469
History of stroke	2 (2.8%)	7 (11.5%)	0.100
History of PAD	5 (6.9%)	4 (6.6%)	1.000
Current smoker	11 (15.5%)	8 (12.9%)	0.81

Procedural variables are presented in Table 2. Radial access was the predominant access site in both groups, with no significant difference in the method of arterial access between the groups. The proportion of patients transferred from an outside hospital was similar between groups. Rates of cardiac arrest prior to arrival in the catheterization laboratory, ventricular tachycardia or fibrillation prior to procedure start, and cardiac arrest prior to or during the procedure were also comparable between groups (*p* > 0.05).

**Table 2:** Study groups case mixture.

Variable	EggNest Complete	Standard Shielding	p-value
Transfer from another hospital	3 (4.2%)	2 (3.3%)	1.000
Cardiac arrest prior to arrival in cath lab	10 (13.9%)	5 (8.2%)	0.448
Ventricular arrythmia prior to arrival in cath lab	7 (9.7%)	2 (3.3%)	0.178
Cardiac arrest in cath lab	4 (5.6%)	2 (3.3%)	0.687
Cardiogenic shock prior to starting case	13 (18.1%)	8 (13.1%)	0.589
Mechanical ventilation during case	16 (22.2%)	11 (18.0%)	0.702
Vasopressor use during case	13 (18.1%)	9 (14.8%)	0.782
MCS use prior to leaving cath lab	7 (9.7%)	6 (9.8%)	1.000
Arterial access site			
Radial	53 (73.6%)	37 (60.7%)	0.160
Femoral	19 (26.4%)	24 (39.3%)	0.160
Multivessel CAD on angiography	26 (36.1%)	30 (49.2%)	0.179
Culprit lesion location			
LAD	27 (37.5%)	24 (39.3%)	0.969
RCA	29 (40.3%)	26 (42.6%)	0.923
LCX	16 (22.2%)	10 (16.4%)	0.532
LM	0 (0.0%)	1 (1.6%)	0.459
D2B time	48.0 [33.0–72]	52.0 [36.0–77.0]	0.32

Similarly, there were no significant differences between groups in the prevalence of cardiogenic shock at presentation, vasopressor use, or use of mechanical circulatory support. The prevalence of multivessel coronary artery disease was comparable, and the distribution of culprit vessels—including left anterior descending, right coronary artery, circumflex, and left main coronary artery—did not differ significantly between groups. Rates of mechanical ventilation prior to or during the procedure were also similar. Median door-to-device (D2B) time did not differ significantly between the EggNest Complete and standard shielding groups (Table 2). Across both cohorts, D2B times were consistent with contemporary guideline-recommended targets.

**Fig. 1:**
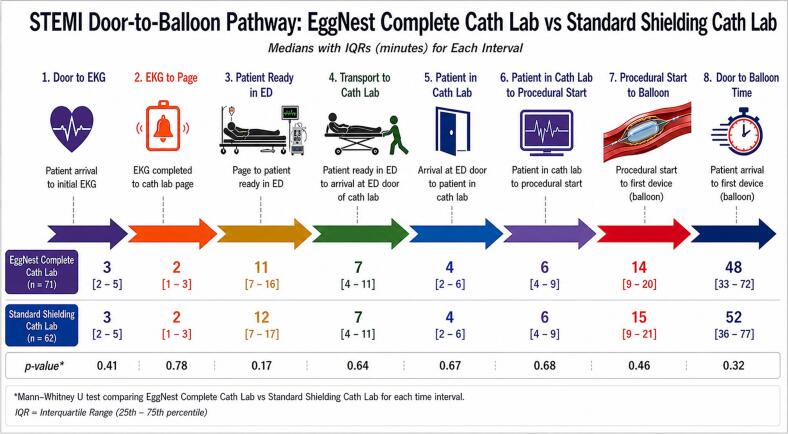
Door-to-balloon times between study groups.

A stepwise analysis of the STEMI workflow is illustrated in Fig. 1, demonstrating comparable time intervals between key stages of care, including door-to-electrocardiogram, activation, patient preparation, transport, and procedural initiation. No individual component of the workflow showed a meaningful delay associated with use of the EggNest Complete ERPD, including the times of patient in CCL to procedural start nor procedure start to balloon times, indicating that use of the EggNest Complete ERPD did not significantly delay treatment times. In a multivariable linear regression model, there was no significant association between procedure room assignment arterial access site, presence of cardiogenic shock, nor need for intubation with D2B time. Within the EggNest Complete group, there was a statistically significant difference in total D2B times in patients based on radial (median 70 min [47.5, 84]) versus femoral (median 87 min [81, 107]) access (*p* < 0.01), likely reflecting clinical practice of radial arterial access preference for STEMI cases with a switch to femoral based on case complexity. It does however show that there is not an increase in DTB times when using the EggNest Complete system when adding in a radial board to the system. Additionally, in the EggNest Complete group, the Spearman correlation for D2B over time was ρ = −0.114 (p 0.035), indicating no significant difference in D2B times between cases done early on during the experience of incorporating the radiation protection device into the STEMI workflow and those cases done later in time, indicating that there was not a learning curing in using the device (Table 3).

**Table 3:** Univariate and multivariable linear regression analysis variables.

Variable	Univariate p-value	β Coefficient (min)	95% CI	Multivariable p-value
EGG vs Non-EGG STEMI	0.188	1.5	−14.1 to 17.1	0.848
Sex (Male vs Female)	0.002	−14.6	−34.0 to 4.8	0.138
Age (per year)	0.062	0.14	−0.26 to 0.53	0.493
Prior CABG	0.183	−0.1	−38.4 to 38.2	0.995
Diabetes Mellitus	0.360	5.7	−12.5 to 23.8	0.539
Out-of-Hospital Cardiac Arrest	0.838	–	–	0.616
In-Hospital VF/Cardiac Arrest Prior to Procedure	0.376	–	–	0.649
VF/Cardiac Arrest During Procedure	0.909	–	–	0.891
Access Site (Radial vs Femoral)	0.029	–	–	0.789
Pre-Procedural Cardiogenic Shock	0.200	–	–	0.187
Vasopressor Use	0.730	–	–	0.592
Mechanical Circulatory Support	0.985	–	–	0.433

Outcome: Door-to-device time (minutes).

Abbreviations: CABG = coronary artery bypass grafting; VF = ventricular fibrillation; STEMI = ST-segment elevation myocardial infarction.

## 4 Discussion

In this study, we evaluated the impact of implementing the EggNest Complete ERPD within the CCL on D2B times in patients presenting with STEMI. The principal finding is that incorporation of this ERPD into routine clinical workflow was not associated with a significant meaningful delay in reperfusion times. Despite the theoretical concern that additional equipment and setup requirements could prolong treatment intervals, D2B times remained comparable between groups, supporting the feasibility of integrating advanced radiation protection technologies into time-sensitive STEMI care.

Given that timely reperfusion remains the cornerstone of STEMI management, any modification to established CCL workflows must be carefully evaluated to ensure that efficiency is not compromised. ERPD platforms introduce structural and ergonomic changes that may initially raise concerns regarding patient access, device manipulation, and procedural setup. However, our findings suggest that these systems can be incorporated without adversely affecting key time intervals in the STEMI care pathway, similar to prior studies evaluating other ERPDs [17]. This likely reflects both operator adaptation and the ability to integrate ERPDs into existing protocols without disrupting established processes.

Importantly, the absence of delay in D2B time must be considered in the context of the substantial occupational benefits associated with ERPDs. Radiation exposure remains a significant long-term hazard for interventional cardiologists and catheterization laboratory staff, with documented risks including malignancy, cataracts, and orthopedic injury related to prolonged use of lead-based protection. Enhanced radiation protection systems have been shown to significantly reduce operator radiation exposure while mitigating the musculoskeletal burden associated with traditional wearable lead aprons. The new “ALARA+” standard mandating incorporation of ERPDs into CCLs underscores the importance of reducing radiation and orthopedic risks of the CCL for the entire CCL team [18]. Our findings suggest that these benefits can be achieved without compromising the timeliness of life-saving reperfusion therapy, addressing a key barrier to broader adoption.

From a workflow perspective, the stepwise analysis of the D2B pathway provides additional insight into where delays might theoretically occur. Although ERPDs implementation could be expected to influence early procedural phases—such as patient transfer, laboratory setup, or vascular access—no significant prolongation was observed across these intervals. This supports the concept that system-level efficiencies and team familiarity can offset any incremental complexity introduced by new technology. It also underscores the importance of multidisciplinary coordination and standardized protocols in maintaining rapid treatment times.

These findings have important implications for care in the CCL. As the field continues to evolve toward improved CCL team safety and ergonomics, there is a need to balance these advancements with the imperative for rapid, high-quality patient care. Our results suggest that these goals are not mutually exclusive and that ERPDs can be adopted without compromising critical performance metrics in STEMI care.

### 4.1 Limitations

This study should be interpreted in light of several limitations. First, as an observational analysis, it is subject to potential selection bias and unmeasured confounding. Second, the study reflects the experience of a single center with established STEMI protocols, which may limit generalizability to institutions with different workflows or levels of experience with different ERPDs. Third, temporal trends and operator learning curves may have influenced the observed results, particularly during the early phases of system implementation. Fourth, we did not include real time scatter radiation monitoring during this study as these measurements are not routinely entered into the medical record. Finally, while D2B time is an important quality metric, this study was not powered to assess clinical outcomes such as mortality or major adverse cardiovascular events.

## 5 Conclusions

Incorporation of the EggNest Complete ERPD into the CCL was not associated with a delay in D2B times among patients undergoing primary PCI for STEMI. These findings support the integration of advanced radiation safety technologies into routine CCL practice, suggesting that improvements in CCL team safety can be achieved without compromising the timeliness of critical reperfusion therapy.

## CRediT authorship contribution statement

**Jennifer Hutchings:** Writing – review & editing, Methodology, Data curation, Conceptualization. **Eve Mai:** Writing – review & editing, Methodology, Data curation, Conceptualization. **Stephen P. Blay:** Methodology, Data curation, Conceptualization. **Chelsea Ballinger:** Writing – review & editing, Methodology, Data curation, Conceptualization. **Robert F. Riley:** Writing – review & editing, Writing – original draft, Visualization, Validation, Supervision, Software, Resources, Project administration, Methodology, Investigation, Funding acquisition, Formal analysis, Data curation, Conceptualization.

## Disclosures statement

RFR serves as an advisor for Egg Medical.

## Ethical statement

IRB approval was obtained for this study by an outside IRB.

## Funding statement

No funding was provided for the design nor completion of this study.

## Declaration of competing interest

The authors declare the following financial interests/personal relationships which may be considered as potential competing interests: Robert F. Riley reports financial support was provided by Egg Medical Inc. Robert F. Riley reports a relationship with Egg Medical Inc that includes: consulting or advisory and equity or stocks. If there are other authors, they declare that they have no known competing financial interests or personal relationships that could have appeared to influence the work reported in this paper.
